# Prediction of perioperative myocardial infarction/injury in high-risk patients after noncardiac surgery

**DOI:** 10.1093/ehjacc/zuad090

**Published:** 2023-08-07

**Authors:** Rebecca Meister, Christian Puelacher, Noemi Glarner, Danielle Menosi Gualandro, Henrik A Andersson, Mirjam Pargger, Gabrielle Huré, Georgiana Virant, Daniel Bolliger, Andreas Lampart, Luzius Steiner, Reka Hidvegi, Giovanna Lurati Buse, Christoph Kindler, Lorenz Gürke, Edin Mujagic, Stefan Schaeren, Martin Clauss, Didier Lardinois, Angelika Hammerer-Lercher, Michelle Chew, Christian Mueller, for the BASEL-PMI Investigators, Katharina Rentsch, Esther Seeberger, Silvia Maiorano, Samantha Weder, Jeanne du Fay de Lavallaz, Ketina Arslani, Andreas Buser, Ivo Strebel, Thomas Wolff, Thomas Nestelberger, Philip Haaf, Murat Bilici, Pedro Lopez Ayala Lopez, Luca Koechlin

**Affiliations:** Cardiovascular Research Institute Basel (CRIB) and Department of Cardiology, University Hospital Basel, University of Basel, Spitalstrasse 2, CH-4031 Basel, Basel-Stadt, Switzerland; Cardiovascular Research Institute Basel (CRIB) and Department of Cardiology, University Hospital Basel, University of Basel, Spitalstrasse 2, CH-4031 Basel, Basel-Stadt, Switzerland; Department of Internal Medicine, University Hospital Basel, University of Basel, Spitalstrasse 21, 4031 Basel, Basel-Stadt, Switzerland; Cardiovascular Research Institute Basel (CRIB) and Department of Cardiology, University Hospital Basel, University of Basel, Spitalstrasse 2, CH-4031 Basel, Basel-Stadt, Switzerland; Cardiovascular Research Institute Basel (CRIB) and Department of Cardiology, University Hospital Basel, University of Basel, Spitalstrasse 2, CH-4031 Basel, Basel-Stadt, Switzerland; Interdisciplinary Medicine in Cardiology Unit, Cardiology Department, Heart Institute (InCor), University of Sao Paulo Medical School, Av. Dr. Enéas Carvalho de Aguiar, 44, Cerqueira César, 05403-900 São Paulo, Brazil; Department of Anaesthesiology and Intensive Care Medicine, Linköping University Hospital, SE-581 83 Linköping, Sweden; Cardiovascular Research Institute Basel (CRIB) and Department of Cardiology, University Hospital Basel, University of Basel, Spitalstrasse 2, CH-4031 Basel, Basel-Stadt, Switzerland; Cardiovascular Research Institute Basel (CRIB) and Department of Cardiology, University Hospital Basel, University of Basel, Spitalstrasse 2, CH-4031 Basel, Basel-Stadt, Switzerland; Cardiovascular Research Institute Basel (CRIB) and Department of Cardiology, University Hospital Basel, University of Basel, Spitalstrasse 2, CH-4031 Basel, Basel-Stadt, Switzerland; Department of Anaesthesiology, University Hospital Basel, University of Basel, Spitalstrasse 21, 4031 Basel, Basel-Stadt, Switzerland; Department of Anaesthesiology, University Hospital Basel, University of Basel, Spitalstrasse 21, 4031 Basel, Basel-Stadt, Switzerland; Department of Anaesthesiology, University Hospital Basel, University of Basel, Spitalstrasse 21, 4031 Basel, Basel-Stadt, Switzerland; Department of Clinical Research, University Basel, Spitalstrasse 21, 4031 Basel, Basel-Stadt, Switzerland; Cardiovascular Research Institute Basel (CRIB) and Department of Cardiology, University Hospital Basel, University of Basel, Spitalstrasse 2, CH-4031 Basel, Basel-Stadt, Switzerland; Department of Anaesthesiology, Cantonal Hospital St. Gallen, Rorschacher Str. 95/Haus 03, 9007 St. Gallen, Switzerland; Department of Anaesthesiology, University Hospital Dusseldorf, Moorenstr. 5 40225 Düsseldorf, NRW, Germany; Department of Anaesthesiology, Cantonal Hospital Aarau, Tellstrasse 25, 5001 Aarau, Aargau, Switzerland; Department of Vascular Surgery, University Hospital Basel, University Basel, Spitalstrasse 21, 4031 Basel, Basel-Stadt, Switzerland; Department of Vascular Surgery, University Hospital Basel, University Basel, Spitalstrasse 21, 4031 Basel, Basel-Stadt, Switzerland; Department of Spinal Surgery, University Hospital Basel, University Basel, Spitalstrasse 21, 4031 Basel, Basel-Stadt, Switzerland; Department of Orthopedics and Center of Musculoskeletal Infections, University Hospital Basel, University Basel, Spitalstrasse 21, 4031 Basel, Basel-Stadt, Switzerland; Department of Thoracic Surgery, University Hospital Basel, University of Basel, Spitalstrasse 21, 4031 Basel, Basel-Stadt, Switzerland; Department of Laboratory Medicine, Cantonal Hospital Aarau, Tellstrasse 25, 5001 Aarau, Aargau, Switzerland; Department of Anaesthesiology and Intensive Care Medicine, Linköping University Hospital, SE-581 83 Linköping, Sweden; Cardiovascular Research Institute Basel (CRIB) and Department of Cardiology, University Hospital Basel, University of Basel, Spitalstrasse 2, CH-4031 Basel, Basel-Stadt, Switzerland

**Keywords:** Troponin, High-sensitivity troponin, Perioperative care, Preoperative care, Perioperative myocardial injury, Diagnostic screening programme

## Abstract

**Aims:**

Perioperative myocardial infarction/injury (PMI) is a surprisingly common yet difficult-to-predict cardiac complication in patients undergoing noncardiac surgery. We aimed to assess the incremental value of preoperative cardiac troponin (cTn) concentration in the prediction of PMI.

**Methods and results:**

Among prospectively recruited patients at high cardiovascular risk (age ≥65 years or ≥45 years with preexisting cardiovascular disease), PMI was defined as an absolute increase in high-sensitivity cTnT (hs-cTnT) concentration of ≥14 ng/L (the 99th percentile) above the preoperative concentration. Perioperative myocardial infarction/injury was centrally adjudicated by two independent cardiologists using serial measurements of hs-cTnT. Using logistic regression, three models were derived: Model 1 including patient- and procedure-related information, Model 2 adding routinely available laboratory values, and Model 3 further adding preoperative hs-cTnT concentration. Models were also compared vs. preoperative hs-cTnT alone. The findings were validated in two independent cohorts. Among 6944 patients, PMI occurred in 1058 patients (15.2%). The predictive accuracy as quantified by the area under the receiver operating characteristic curve was 0.73 [95% confidence interval (CI) 0.71–0.74] for Model 1, 0.75 (95% CI 0.74–0.77) for Model 2, 0.79 (95% CI 0.77–0.80) for Model 3, and 0.74 for hs-cTnT alone. Model 3 included 10 preoperative variables: age, body mass index, known coronary artery disease, metabolic equivalent >4, risk of surgery, emergency surgery, planned duration of surgery, haemoglobin, platelet count, and hs-cTnT. These findings were confirmed in both independent validation cohorts (*n* = 722 and *n* = 966).

**Conclusion:**

Preoperative cTn adds incremental value above patient- and procedure-related variables as well as routine laboratory variables in the prediction of PMI.

## Graphical Abstract

**Graphical Abstract:**
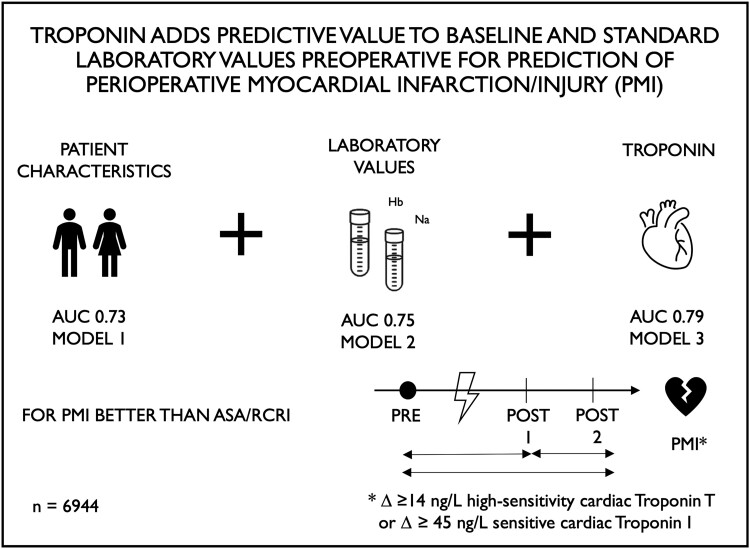
ASA, American society of anesthesiology; AUC, area under the receiver operating characteristics curve; Hb, haemoglobin; Na, potassium; RCRI, revised cardiac risk index

## Introduction

With over 300 million surgeries performed annually, perioperative complications are of major medical and economic relevance.^1–4^ Despite important advances in surgical and anaesthesiologic techniques, perioperative mortality remained higher than commonly anticipated.^5^ Perioperative myocardial infarction/injury (PMI) has recently been identified as a common yet still incompletely understood cardiac complication following noncardiac surgery and an important contributor to perioperative mortality.^1–4^ Current 2022 European Society of Cardiology (ESC) and European Society of Anaesthesiology clinical practice guidelines advocate with a class I recommendation active surveillance for PMI in high-risk patients.^2,6^ In order to target PMI active surveillance efforts to patients at highest risk, accurate and widely available prediction tools for PMI would be desirable but are currently lacking.^7^

Accurate estimation of the risk of PMI as the most common cardiac complication following noncardiac surgery would be a requirement for joined informed decision-making among physicians and patients regarding the risk–benefit ratio of the planned operation, for identifying the possible need for additional preoperative cardiac workup, possibly a less invasive surgical approach, and/or more intense or more prolonged perioperative monitoring.^3,4^ It is conceivable that established risk prediction tools developed to predict perioperative death such as the American Society of Anesthesiologists (ASA classification) physical status classification and the revised cardiac risk index (RCRI) also allow the prediction of PMI.^2,8–13^

Therefore, the aim of this prospective study was first to evaluate the predictive accuracy of the ASA classification and the RCRI for PMI, second, to derive multivariable prediction models that reflect different clinical real-life scenarios using routine variables and evaluating the addition of cardiac troponin (cTn), third, to directly compare the ASA classification and the RCRI with the derived prediction models, and fourth, to assess the performance of preoperative cTn concentration as a single variable.

## Methods

We adhered to the transparent reporting of a multivariable prediction model for individual prognosis or diagnosis Statement for reporting our findings.^14^

### Study design

This was a prespecified analysis of the prospective multicentre Basel-PMI study (NCT02573532). We included consecutive patients from October 2014 to February 2018 who provided written general consent to registration in a dedicated prospective database.^3^ This study was approved by the local ethics committee.

### Patients

We included consecutive patients undergoing noncardiac surgery at the University Hospital Basel, Switzerland, who were eligible for their institutional routine PMI active surveillance programme. Patients were included if they had a planned hospital stay exceeding 24 h after surgery and were considered at increased risk of mortality, defined as ≥65 years of age or ≥45 years with a history of coronary artery disease (CAD), peripheral artery disease (PAD), or stroke. Monitoring was implemented for patients undergoing visceral, orthopaedic, trauma, vascular, urologic, spinal, and thoracic surgical procedures.^3^ Patients were excluded if PMI could not be reliably diagnosed, for example, due to missing pre- or postoperative cTn measurements.

### Perioperative myocardial infarction/injury prediction models

The ASA classification and the RCRI were constructed as recommended in current clinical practice guidelines (see Supplementary material online, *Tables S1* and S*2*). In order to best reflect different clinical real-life scenarios, three multivariable prediction models for PMI were derived: Model 1 based on clinical information obtainable at the bedside, Model 2 adding routinely available laboratory measurements such as haemoglobin and platelet count, and Model 3, further adding high-sensitivity cTnT (hs-cTnT) given its strong predictive power for perioperative death.^15,16^

### Laboratory measurements

Standard laboratory measurements were collected from preoperative routine blood counts and blood chemistries. We restricted the laboratory values in the model to those routinely available preoperatively that have previously been found to be predictive of outcome and were also available in at least 80% of patients in this cohort. We categorized each parameter into three groups according to commonly used lower and upper reference values.^17–19^ Haemoglobin was classified into normal (≥130 g/L for men and ≥120 g/L for women), mild to moderate anaemia (80–130 g/L for men and 80–120 g/L for women), and severe anaemia (<80 g/L). Platelets were categorized into normal (150–450 × 10^9^/L), thrombopaenia (<150 × 10^9^/L), and thrombocytosis (>450 × 10^9^/L). Leucocytes were divided into normal (4–10 × 10^9^/L), leucopoenia (<4 × 10^9^/L), and leucocytosis (>10 × 10^9^/L). Sodium was subdivided into normal (135–145 mmol/L), hyponatraemia (<135 mmol/L), and hypernatraemia (>145 mmol/L). Potassium was classified as normal (3.6–5.2 mmol/L), hypokalaemia (<3.6 mmol/L), and hyperkalaemia (>5.2 mmol/L).

Plasma concentrations of hs-cTnT were measured within 30 days before surgery, on postoperative Days 1 and 2, and later if clinically indicated. High-sensitivity cardiac troponin T was measured on the Modular Analytics E170 or the Cobas e602 (Roche Diagnostics) assay with a limit of detection of 5 ng/L, a 10% coefficient of variation at 13 ng/L, and the 99th percentile of a healthy reference population at 14 ng/L.^3^

### Endpoints

Perioperative myocardial infarction/injury was prospectively defined as an absolute increase of ≥14 ng/L for hs-cTnT and ≥45 ng/L for sensitive cardiac troponin I (s-cTnI) (the 99th percentile of each assays) above the preoperative concentration (or between two postoperative concentrations if the preoperative measurement was missing) within 3 days following surgery. Based on consistent evidence from prior studies that PMI is associated with increased mortality irrespective of the presence of additional symptoms and signs used for the definition of spontaneous acute myocardial infarction (AMI), the definition of PMI was based on acute perioperative elevation of cTn alone as recommended by current guidelines (examples provided in Supplementary material online).^2–4^ We used delta values instead of maximum postoperative concentrations to ensure that our definition reflected acute myocardial damage and was time related to surgery, thus avoiding misclassification of chronically elevated levels.^3,15,20^ We chose an absolute rather than a relative delta hs-cTnT level for the diagnosis of PMI, because absolute changes have shown higher diagnostic accuracy than relative changes in the detection of AMI in the nonoperative setting.^21,22^ The absolute increase of +≥14 ng/L was selected as it represents the 99th percentile of healthy individuals, and, thereby, all PMIs invariably would fulfil the change, and the absolute cTn criteria, as well, required for the diagnosis of spontaneous AMI.^3,23^ Perioperative myocardial infarction/injury was centrally adjudicated including PMI subclassification by two independent experts based on all clinical information obtained during the index hospitalization, including electrocardiogram, serial laboratory measurements including hs-cTnT and haemoglobin, monitoring of vital signs in the perioperative and intraoperative period, echocardiography, cardiac stress testing, and coronary angiography, if performed. In cases of disagreement between the two reviewers, consensus was sought and found by discussion with a third senior physician.

As a *post hoc* analysis, we also calculated the predictive accuracy of Model 3, preoperative hs-cTnT, RCRI, and ASA classification for ‘myocardial injury after noncardiac surgery (MINS)’. Myocardial injury after noncardiac surgery represents a subgroup of PMI that was considered to be related to CAD and that fulfiled hs-cTnT criteria derived in the VISION Study^4^: a postoperative hs-cTnT concentration of 20 to <65 ng/L with an absolute change of at least 5 ng/L or hs-cTnT concentration ≥ 65 ng/L.^23^

### Statistical analysis

All data analysis was performed on SPSS 26.0 (IBM Corp., Armonk, NY). Numerical variables are presented as medians and interquartile range while categorical variables are reported as frequencies (percentages). Baseline characteristics were compared by using the *χ*^2^ or Mann–Whitney test as appropriate. *P* values of <0.05 were considered to be statistically significant. We constructed three models incorporating an increasing amount of variables representing increasing amounts of preoperative information. For our multivariable binary logistic regression modelling for the prediction of PMI, we chose forced entry including variables identified *via* literature research and predefined cardiovascular risk factors.^16,24–26^ We chose to include a maximum of 1 variable per 10 events into our regression analysis.^27^ We checked the variables for collinearity by testing the correlation coefficient with Pearson’s correlation for numerical variables, Phi and Cramer’s *V* for nominal variables, and Spearman’s correlation for categorical variables and also by calculating the variance inflation factor (VIF). We performed the multivariable logistic regression stepwise in three models: In the first model (Model 1), preoperative available clinical or surgical variables were included. We used age, sex, body mass index (BMI), history of hypertension, diabetes, CAD or previous AMI, heart failure, atrial fibrillation, PAD, previous transient ischaemic attack or stroke, and chronic kidney disease; metabolic equivalent of task (MET) with a cut-off of 4^28^; the risk of surgery according to the ESC clinical practice guidelines^2^; the urgency of the surgery; preoperative systolic blood pressure; and the planned duration of the surgery. Variables not meeting the predefined significance level (*P* < 0.05) were omitted. In the second model (Model 2), we added on top of variables from Model 1 haemoglobin, platelets, leucocytes, sodium, and potassium to the clinical parameters. Variables not meeting the predefined significance level (*P* > 0.05) were omitted. In the third approach (Model 3), we added preoperative hs-cTnT on top of Model 2.

To compare the additional value added in each modelling step, we compared the models by calculating the area under the receiver operating characteristic curve (AUC) for each model and comparing the AUCs with their 95% confidence interval (CI), as well as calculating the Akaike information criterion (AIC). Further, the models and cTn were compared with ASA and RCRI.

### Recalibration for sensitive cardiac troponin I

The derived model from the main cohort was recalibrated for s-cTnI in the BASEL-PMI population of another centre (Cantonal Hospital Aarau, Switzerland), which had identical inclusion criteria as the main cohort but routinely measured s-cTnI. In this cohort, PMI was adjudicated using s-cTnI, using the respective 99th percentile of 45 ng/L to define the required absolute increase above preoperative s-cTnI concentrations. We had to exclude the planned duration of surgery as a variable, because this parameter was not recorded in the database of this study centre. Sensitive cardiac troponin I was measured using a Siemens Dimension Vista assay (Siemens Health Care Diagnostics, Tarrytown, NY). This s-cTnI assay has a limit of detectionof 15 ng/L and a 99th percentile of 45 ng/L.^29^

### Validation

To assess the external validity of our model, we validated the findings of the hs-cTnT Model 3 in an independent cohort enroled in Sweden [Myocardial Injury in Noncardiac Surgery in Sweden (MINSS), NCT03436238], which overall used comparable inclusion criteria but exclusively enroled patients undergoing elective major abdominal surgery.

## Results

**Figure 1:**
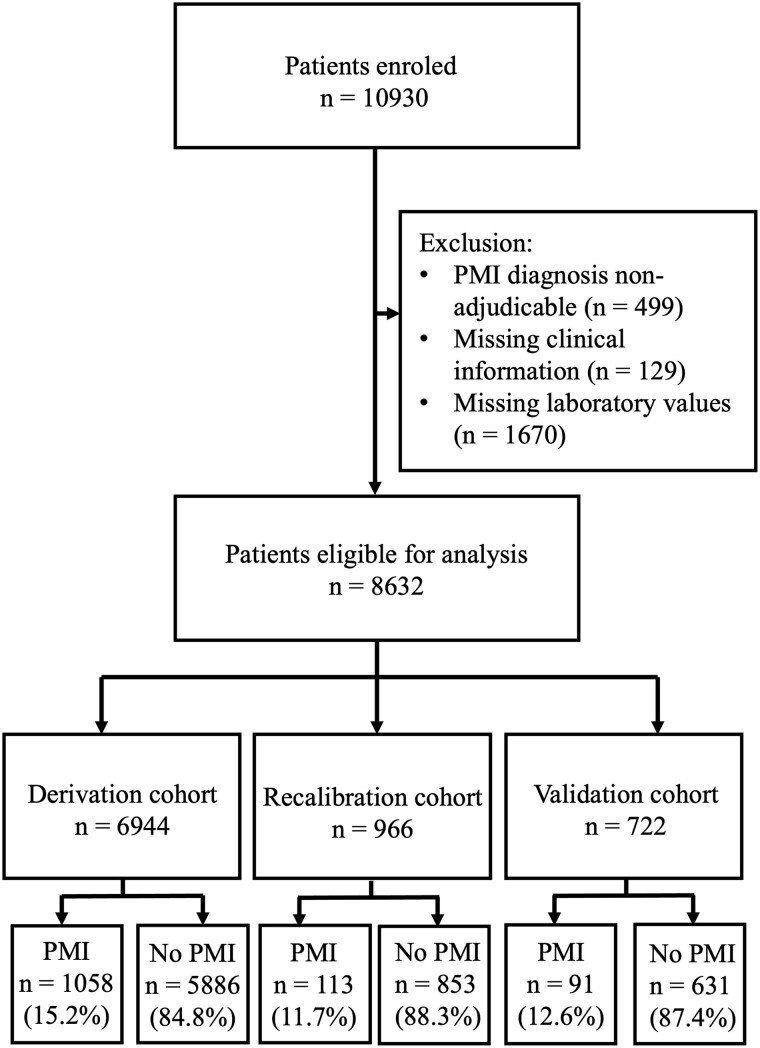
Patient flow chart. Study population selection can be seen in this flow chart. Missing laboratory values included missing preoperative routinely available laboratory values, as well as missing preoperative hs-cTnT or s-cTnI values. PMI, perioperative myocardial infarction/injury; hs-cTnT, high-sensitivity cardiac troponin T; s-cTnI, sensitive cardiac troponin I.

Among the 8632 patients enroled from October 2014 to February 2018 who were eligible for this analysis, 6944 patients were assigned to the derivation cohort, 966 to the recalibration cohort, and 722 to the validation cohort (*Figure 1*; Supplementary material online, *Table S3*).

In the derivation cohort, the median age was 74 years, 43.3% were women, 30.5% had known CAD, 20.2% PAD, and 18.5% atrial fibrillation (*Table 1*). The operations most commonly performed were within orthopaedics (20.4%), spinal surgery (16.9%), urology (16.3%), vascular surgery (14.9%), visceral surgery (12.3%), thoracic surgery (9.3%), and traumatology (7.6%). Perioperative myocardial infarction/injury occurred in 1058 patients (15.2%).

**Table 1:** Baseline characteristics of the derivation cohort

	All patients (*n* = 6944; 100%)	PMI (*n* = 1058; 15.2%)	No PMI (*n* = 5886; 84.8%)	*P* value
Age, years, median (IQR)	74 (69–79)	76 (70–81)	74 (68–79)	<0.001
Male, sex, *n* (%)	3937 (56.7%)	634 (59.9%)	3303 (56.1%)	0.021
BMI, kg/m^2^, *n* (%)				0.002
<18.5	242(3.5%)	48 (4.5%)	194 (3.3%)	
18.5–24.9	2706 (39.0%)	449 (42.4%)	2257 (38.3%)	
≥25	3996 (57.5%)	561 (53.0%)	3435 (58.4%)	
Risk factors, *n* (%)				
Hypertension	4603 (66.3%)	770 (72.8%)	3833 (65.1%)	<0.001
Diabetes				<0.001
NIDDM	1064 (15.3%)	168 (15.9%)	896 (15.2%)	
IDDM	662 (9.5%)	166 (15.7%)	496 (8.4%)	
Medical history, *n* (%)				
Coronary artery disease	2118 (30.5%)	487 (46.0%)	1631 (27.7%)	<0.001
Previous myocardial infarction	1075 (15.5%)	274 (25.9%)	801 (13.6%)	<0.001
Congestive heart failure				<0.001
Preserved LVEF (>50%)	253 (3.6%)	75 (7.1%)	178 (3%)	
Medium LVEF (40–50%)	247 (3.6%)	69 (6.5%)	178 (3%)	
Reduced LVEF (<40%)	302 (4.3%)	84 (7.9%)	218 (3.7%)	
LVEF not graduated	36 (0.5%)	8 (0.8%)	28 (0.5%)	
Atrial fibrillation	1287 (18.5%)	302 (28.5%)	985 (16.7%)	<0.001
Peripheral artery disease	1406 (20.2%)	277 (26.2%)	1129 (19.2%)	<0.001
Previous stroke or TIA	684 (9.9%)	133 (12.6%)	551 (9.4%)	0.001
Chronic kidney disease				<0.001
CKD I–II	2246 (32.3%)	357 (33.7%)	1889 (32.1%)	
CKD III+	1023 (14.7%)	235 (22.2%)	788 (13.4%)	
Dialysis-dependent CKD	132 (1.9%)	54 (5.1%)	78 (1.3%)	
Functional capacity > 4 METS	3609 (52.0%)	354 (33.5%)	3255 (55.3%)	<0.001
Type of surgery, *n* (%)				<0.001
Orthopaedic	1419 (20.4%)	218 (20.6%)	1201 (20.4%)	
Spinal	1173 (16.9%)	171 (16.2%)	1002 (17.0%)	
Thoracic	644 (9.3%)	163 (15.4%)	481 (8.2%)	
Trauma	526 (7.6%)	96 (9.1%)	430 (7.3%)	
Urologic	1131 (16.3%)	108 (10.2%)	1023 (17.4%)	
Visceral	856 (12.3%)	96 (9.1%)	760 (12.9%)	
Vascular	1036 (14.9%)	189 (17.9%)	847 (14.4%)	
Other	159 (2.3%)	17 (1.6%)	142 (2.4%)	
Risk of surgery, *n* (%)				<0.001
Low (<1%)	2255 (32.5%)	227 (21.5%)	2028 (34.5%)	
Intermediate (1–5%)	3908 (56.3%)	656 (62.0%)	3252 (55.2%)	
High (>5%)	781 (11.2%)	175 (16.5%)	606 (10.3%)	
Time of surgery, *n* (%)				<0.001
Elective	5055 (72.8%)	703 (66.4%)	4352 (73.9%)	
Emergency < 24 h	648 (9.3%)	134 (12.7%)	514 (8.7%)	
Emergency > 24 h	1241 (17.9%)	221 (20.9%)	1020 (17.3%)	
Other parameters, median (IQR**)**				
Preoperative SBP, mmHg	136 (123–150)	135 (120–150)	137 (123–150)	0.023
Planned duration of surgery, min	135 (105–165)	165 (105–195)	135 (105–165)	<0.001
Risk score ASA, *n* (%)				<0.001
1	72 (1.0%)	2 (0.2%)	70 (1.2%)	
2	1988 (28.6%)	145 (13.7%)	1843 (31.3%)	
3	4371 (62.9%)	749 (70.8%)	3622 (61.5%)	
4	509 (7.3%)	160 (15.1%)	349 (5.9%)	
5	4 (0.1%)	2 (0.2%)	2 (0.0%)	
RCRI, *n* (%)				<0.001
I	2969 (42.8%)	263 (24.9%)	2706 (46.0%)	
II	2285 (32.9%)	363 (34.3%)	1922 (32.7%)	
III	1124 (16.2%)	239 (22.6%)	885 (15.0%)	
IV	566 (8.2%)	193 (18.2%)	373 (6.3%)	
Biochemistry, median (IQR)				
Haemoglobin, g/L	126 (109–140)	113 (95–130)	128 (112–141)	<0.001
Platelets, ×10^9^/L	242 (193–306)	247 (191–338)	242 (194–301)	0.017
Leucocytes, ×10^9^/L	7.5 (6.1–9.4)	8.0 (6.3–10.4)	7.4 (6.0–9.3)	<0.001
Creatinine, µmol/L	81.0 (67.0–105)	91.0 (70.0–128)	80.0 (66.0–101)	<0.001
Sodium, mmol/L	140 (137–141)	139 (137–141)	140 (138–141)	0.167
Potassium, mmol/L	4.1 (3.8–4.4)	4.2 (3.8–4.6)	4.1 (3.8–4.4)	<0.001
Preoperative hs-cTnT, ng/L	14.0 (9.0–26.0)	29.0 (15.0–56.0)	13.0 (8.0–23.0)	<0.001

Baseline characteristics of the study population from the derivation cohort can be seen in this table.

ASA, American Society of Anesthesiologists; BMI, body mass index; CKD, chronic kidney disease; hs-cTnT, high-sensitivity cardiac troponin T; IDDM, insulin-dependent diabetes mellitus; LVEF, left ventricular ejection fraction; MET, metabolic equivalent of task; NIDDM, noninsulin-dependent diabetes mellitus; PMI, perioperative myocardial infarction/injury; RCRI, revised cardiac risk index; SBP, systolic blood pressure; TIA, transient ischaemic attack.

### Prediction of perioperative myocardial infarction/injury

**Figure 2:**
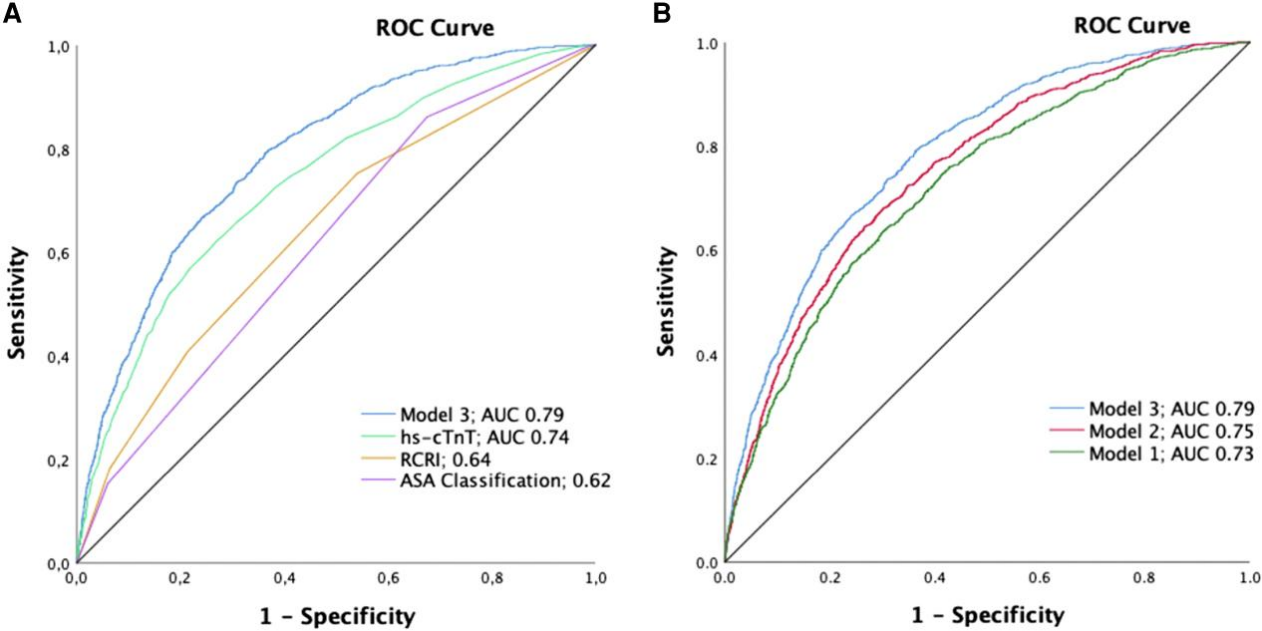
Accuracy for the prediction of perioperative myocardial infarction/injury. Area under the receiver operating characteristic curve to compare the diagnostic accuracy of Model 3 with the RCRI, the ASA classification, and hs-cTnT alone (*A*). Area under the receiver operating characteristic curve quantifying the accuracy of the three prediction models in the derivation cohort (*B*). AUC, area under the receiver operating characteristic curve; RCRI, revised cardiac risk index; ASA, American Society of Anesthesiologists.

The predictive accuracy for PMI as quantified by the AUC was modest for both the ASA classification with 0.62 (95% CI 0.60–0.64) and the RCRI with 0.64 (95% CI 0.62–0.66; *Figure 2A*). The AUC for preoperative hs-cTnT alone as a predictor of PMI was 0.74 (95% CI 0.72–0.75), which was higher compared with ASA (*P* < 0.001) and RCRI (*P* < 0.001). When hs-cTnT was added to each of the two risk scores ASA classification and RCRI, the AUC did not significantly improve the AUC vs. hs-cTnT alone.

### Novel perioperative myocardial infarction/injury prediction models

**Figure 3:**
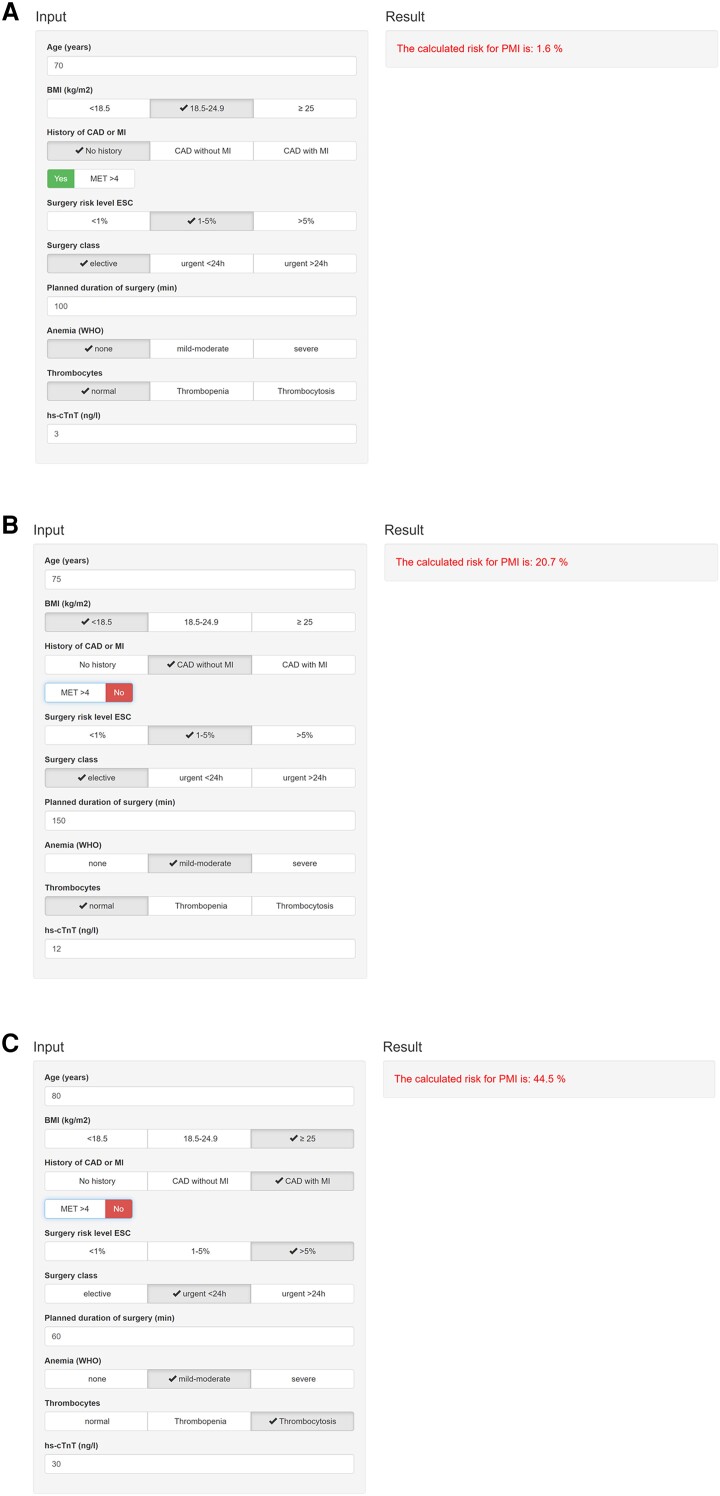
Examples for usage of the online calculator tool. A patient with age of 70 years, normal weight, no history of coronary artery disease or myocardial infarction, metabolic equivalent of task > 4, intermediate risk of surgery according to the European Society of Cardiology, elective surgery, planned duration of surgery of 100 min, no anaemia, normal level of thrombocytes, and a high-sensitivity cardiac troponin T of 3 ng/L has a risk of perioperative myocardial infarction/injury of 1.6% (*A*). A patient with age of 75 years, underweight, history of coronary artery disease without myocardial infarction, metabolic equivalent of task < 4, intermediate risk of surgery according to the European Society of Cardiology, elective surgery, planned duration of surgery of 150 min, mild-to-moderate anaemia, normal level of thrombocytes, and a high-sensitivity cardiac troponin T of 12 ng/L has a risk of perioperative myocardial infarction/injury of 20.7% (*B*). A patient with age of 80 years, overweight, history of coronary artery disease with myocardial infarction, metabolic equivalent of task < 4, high risk of surgery according to the European Society of Cardiology, urgent surgery <24 h, planned duration of surgery of 60 min, mild-to-moderate anaemia, thrombocytosis, and a high-sensitivity cardiac troponin of 30 ng/L has a risk of perioperative myocardial infarction/injury of 44.5% (*C*). PMI, perioperative myocardial infarction/injury; BMI, body mass index; CAD, coronary artery disease; MI, myocardial infarction; MET, metabolic equivalent of task; ESC, European Society of Cardiology; WHO, World Health Organization.

The full models are shown in *Table 2* and Supplementary material online, *Tables S4–S6*. Overall, the predictive accuracy was moderate to high, with an AUC of 0.73 (95% CI 0.71–0.74) for Model 1 including clinical and surgical variables, 0.75 (95% CI 0.74–0.77) for Model 2 including also routinely available laboratory measurements, and 0.79 (95% CI 0.77–0.80) for Model 3 when further adding preoperative hs-cTnT concentrations (*Figure 2B*). The addition of the new variables showed an improvement in comparison to the previous model, with *P* < 0.001 and an improvement in AIC (see Supplementary material online, *Table S7*). The model performing the best was Model 3 (clinical variables with additional preoperative laboratory values and preoperative hs-cTnT) with a correct prediction of 88.2% of all the observed cases. We identified 10 criteria as independent predictors for PMI: age, BMI, CAD with or without previous AMI, MET > 4, risk of surgery, emergency surgery, planned duration of surgery, preoperative haemoglobin, preoperative platelets, and preoperative hs-cTnT. Ln-transformed hs-cTnT was strongly associated with PMI with an odds ratio (OR) of 2.38 (95% CI 2.16–2.62, *P* < 0.001) per ln increase. The AUC for preoperative hs-cTnT alone as a predictor of PMI was 0.74 (95% CI 0.72–0.75). Examples for the use of Model 3 as an online PMI risk calculator (https://pmicalculator.shinyapps.io/Calculator/) are provided in *Figure 3*.

**Table 2:** Logistic regression models predicting perioperative myocardial infarction/injury in the derivation cohort

Variables, OR (95% CI for OR)	Model 1	Model 2	Model 3
Age, years	1.020 (1.010–1.030)	1.022 (1.012–1.033)	1.011 (1.001–1.021)
BMI 18.5–24.9 kg/m^2^			
BMI < 18.5 kg/m^2^	1.266 (0.890–1.801)	1.190 (0.834–1.698)	1.192 (0.829–1.716)
BMI ≥ 25 kg/m^2^	0.755 (0.652–0.875)	0.819 (0.705–0.951)	0.804 (0.692–0.934)
No diabetes			
NIDDM	1.034 (0.850–1.258)	0.962 (0.789–1.173)	
IDDM	1.571 (1.264–1.952)	1.385 (1.111–1.726)	
No history of CAD or MI			
CAD without MI	1.490 (1.230–1.804)	1.455 (1.199–1.766)	1.353 (1.116–1.641)
CAD with MI	2.073 (1.730–2.485)	2.033 (1.692–2.442)	1.776 (1.476–2.137)
History of heart failure	1.318 (1.083–1.605)	1.242 (1.019–1.515)	
AF	1.313 (1.107–1.557)	1.223 (1.028–1.454)	
No CKD			
CKD I–II	1.348 (1.138–1.598)	1.300 (1.095–1.545)	
CKD III+	1.799 (1.473–2.197)	1.621 (1.323–1.987)	
Dialysis-dependent CKD	4.285 (2.893–6.349)	3.834(2.581–5.695)	
MET >4	0.550 (0.474–0.639)	0.650 (0.557–0.758)	0.780 (0.666–0.912)
Low-risk surgery ESC <1%			
Intermediate risk surgery ESC 1–5%	1.582 (1.325–1.889)	1.687 (1.409–2.020)	1.770 (1.470–2.131)
High-risk surgery ESC >5%	1.834 (1.447–2.324)	1.806 (1.421–2.296)	1.804 (1.412–2.304)
Elective surgery			
Urgent surgery <24 h	2.018 (1.604–2.540)	1.781 (1.400–2.266)	1.724 (1.362–2.182)
Urgent surgery >24 h	1.418 (1.180–1.705)	1.169 (0.967–1.414)	1.106 (0.918–1.333)
Planned duration of surgery, min	1.005 (1.004–1.007)	1.006 (1.005–1.007)	1.007 (1.005–1.008)
Hb normal, g/L			
Mild-to-moderate anaemia		2.089 (1.780–2.452)	1.538 (1.302–1.818)
Severe anaemia		2.322 (1.624–3.319)	1.261 (0.886–1.836)
Tc normal, ×10^9^/L			
Thrombopaenia		1.208 (0.951–1.535)	1.061 (0.833–1.351)
Thrombocytosis		1.613 (1.254–2.075)	1.583 (1.230–2.037)
Lc normal, ×10^9^/L			
Leucopoenia		0.733 (0.455–1.179)	
Leucocytosis		1.328 (1.118–1.578)	
Sodium normal, mmol/L			
Hyponatraemia		1.098 (0.876–1.376)	
Hypernatraemia		1.685 (1.126–2.522)	
hs-cTnT (ln), ng/L			2.382 (2.164–2.622)

Prediction of PMI using only clinical information (Model 1), clinical information with additional preoperative routinely available laboratory values (Model 2), and clinical information with additional preoperative routinely available laboratory values and preoperative hs-cTnT (Model 3).

AF, atrial fibrillation; BMI, body mass index; CAD, coronary artery disease; CI, confidence interval; CKD, chronic kidney disease; ESC, European Society of Cardiology; Hb, haemoglobin; hs-cTnT, high-sensitivity cardiac troponin T; IDDM, insulin-dependent diabetes mellitus; Lc, leucocyte; MET, metabolic equivalent of task; MI, myocardial infarction; NIDDM, noninsulin-dependent diabetes mellitus; OR, odds ratio; PMI, perioperative myocardial infarction/injury; Tc, thrombocyte.

### Recalibration for sensitive cardiac troponin I

**Figure 4:**
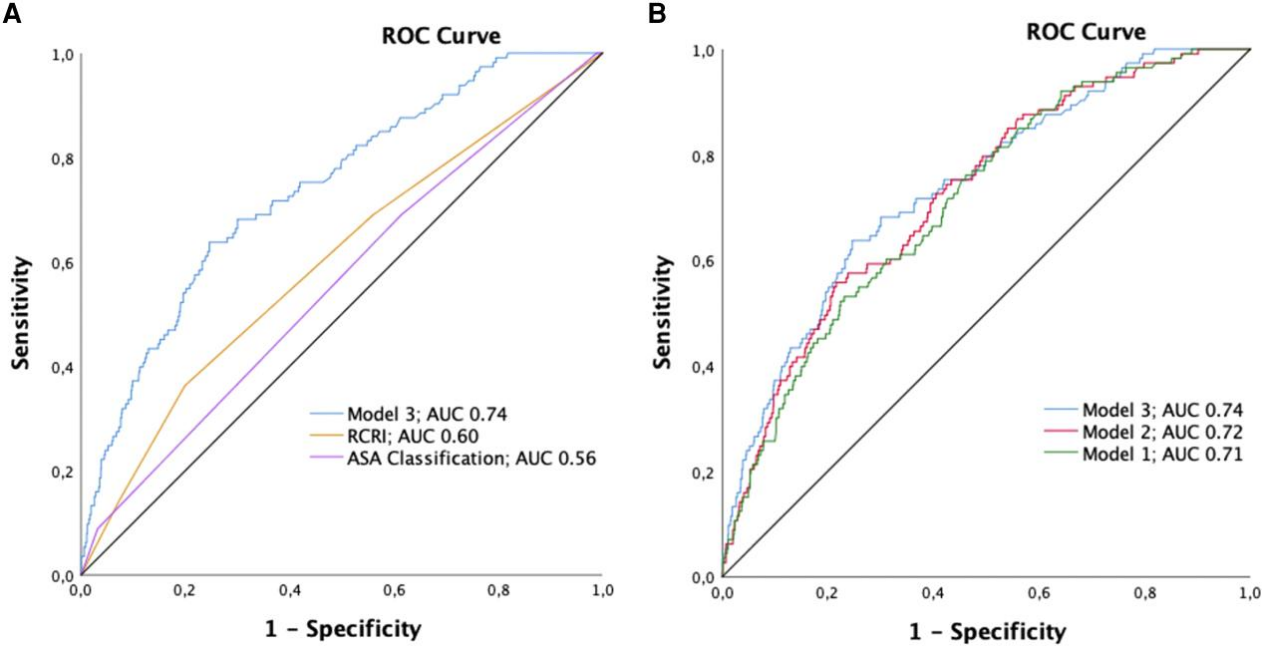
Recalibration cohort. Comparison of Model 3 with the revised cardiac risk index and the American Society of Anesthesiologists classification for the prediction of perioperative myocardial infarction/injury in the recalibration cohort (*A*). Comparison of the area under the receiver operating characteristic curve of the three prediction models to perioperative myocardial infarction/injury in the recalibration cohort (*B*). RCRI, revised cardiac risk index; ASA, American Society of Anesthesiologists; PMI, perioperative myocardial infarction/injury; AUC, area under the receiver operating characteristic curve.

Overall, patient baseline characteristics were comparable in patients enroled in the s-cTnI cohort (see Supplementary material online, *Table S8*). The median age was 74 years, 44.6% were women, and 28.6% had known CAD. Perioperative myocardial infarction/injury developed in 113 patients (11.7%). Only about half of the predictors from the derivation cohort retained statistical significance in the recalibrated model, including s-cTnI (see Supplementary material online, *Table S9*). The AUC of preoperative s-cTnI alone as predictor of PMI was 0.62 (92% CI 0.56–0.68, *P* < 0.001). The predictive accuracy for PMI as quantified by the AUC was modest for the ASA classification with 0.56 (95% CI 0.50–0.61) and the RCRI with 0.60 (95% CI 0.54–0.66) (*Figure 4A*) and moderate to high with 0.71 (95% CI 0.66–0.76) for Model 1, 0.72 (95% CI 0.68–0.77) for Model 2, and 0.74 (95% CI 0.69–0.79) for Model 3 (*Figure 4B*).

### External validation

In the external validation cohort, 722 patients (of 737) had all laboratory values available for analysis (see Supplementary material online, *Table S10*). The median age was 70 years, 43% of patients were women, and 13% of patients had CAD. Perioperative myocardial infarction/injury occurred in 91 patients (12.6%). The AUC for the prediction of PMI was 0.64 (95% CI 0.59–0.70) for Model 3 and 0.63 (95% CI 0.57–0.70) for hs-cTnT alone.

### *Post hoc* analysis prediction of myocardial injury after noncardiac surgery

Myocardial injury after noncardiac surgery occurred in 1967 patients (28.3%). Predictive accuracy of the RCRI and the ASA classification was both modest with an AUC of 0.62 and 0.61, respectively. The AUC of Model 3 was 0.80 and the AUC for preoperative hs-cTnT concentration alone was 0.79 (see Supplementary material online, *Figure S1*).

## Discussion

We prospectively derived and validated prediction models for PMI, a common yet difficult-to-predict cardiac complication in patients undergoing noncardiac surgery in a large cohort of high-risk patients.^3,4,23^ We report four major findings.

First, the ASA classification and the RCRI had only modest predictive accuracy (AUC 0.62 and 0.64) for PMI. Second, a model including all available information concerning patient and surgical characteristics and routine laboratory variables achieved moderate-to-good predictive accuracy (AUC 0.75), which could be further improved by adding preoperative hs-cTnT (AUC 0.79). Third, preoperative hs-cTnT concentration as a single variable also had moderate-to-good predictive accuracy for PMI (AUC 0.74). Fourth, 10 independent predictors for PMI emerged: age, BMI, CAD with or without previous myocardial infarction, MET > 4, risk of surgery, emergency surgery, planned duration of surgery, preoperative haemoglobin, preoperative platelets, and preoperative hs-cTnT concentration. These variables were combined in an online PMI risk calculator to facilitate possible clinical use. Fifth, this PMI prediction model as well as hs-cTnT as a single variable also had moderate-to-high predictive accuracy (AUC 0.80 and 0.79, respectively) for MINS, a PMI subgroup considered to be related to CAD.^4^

These findings corroborate and extend previous research deriving risk indices for patients undergoing noncardiac surgery most commonly using a composite of cardiovascular death and myocardial infarction as the endpoint.^10,12,30–32^ These findings have important clinical implications: Accurate preoperative risk estimation for major perioperative complications is a prerequisite for informed joint decision-making among physicians and patients regarding the risk–benefit ratio of a planned operation. As most elective surgeries are performed for conditions for which surgery is one of the treatment options, but are neither urgent nor mandatory, identification of a high risk for perioperative complications would likely result in an informed joint decision to delay or even completely avoid surgery. In contrast, identification of a low risk for perioperative complications would likely result in an informed joint decision favouring surgery. In addition, identification of a high risk for perioperative complications could also highlight the possible need for an additional preoperative cardiac workup and therapy, which could potentially lower perioperative risk. Alternative clinical approaches when high risk for perioperative cardiac complications is detected include selection of a less invasive surgical approach and/or more intense or more prolonged perioperative monitoring.^3,4^

The identified independent predictors of PMI reflect patient, urgency, and procedure-related variables, highlighting the complex interplay of these three domains in the pathophysiology of perioperative complications. These findings are supported by recent observations that preoperative anaemia independently predicts PMI after noncardiac surgeries.^33,34^ In another study, reduced MET capacity was also independently associated with the occurrence of major adverse cardiac events as well as increased all-cause mortality, improving preoperative risk classification.^28^

Similarly, these findings are supported by prior observations regarding the moderate-to-high predictive accuracy of preoperative hs-cTnT concentrations alone for major perioperative cardiac complications including death, clearly underlining the incremental value of this biomarker.^4,7,35^ A recent systematic review and meta-analysis found preoperative cTn concentration to be a strong and independent predictor of in-hospital/30-day risk of major adverse cardiac events (OR 4.3, 95% CI 2.9–6.5, *P* < 0.001; adjusted OR 5.87, 95% CI 3.24–10.65, *P* < 0.001).^35^ This study also extends and corroborates a prior pilot study indicating possible differences in the predictive value of cTnT vs. cTnI. Future studies including additional hs-cTnI assays need to clarify whether the differences regarding predictive accuracy observed in this study (AUC hs-cTnT 0.74 vs. s-cTnI 0.62) are mainly driven by different predictive values of the respective analyte (cTnT vs. cTnI) or by analytical differences related to the specific cTn assays used.^16^

Our findings also suggest that the predictive accuracy of the newly derived PMI prediction models may differ according to surgical discipline.

### Strengths

To the best of our knowledge, this is the largest PMI prediction study performed to date. It included central adjudication of PMI with preoperative hs-cTnT/I measurements to reliably differentiate PMI from chronic hs-cTnT/I elevations, which are very common in high-risk patients undergoing noncardiac surgery. It also included emergency and urgent surgeries (25.8%), as well as surgeries performed on weekends to fully reflect real-life clinical care. Findings were externally validated in another study cohort.

### Limitations

Several limitations should be considered when interpreting these findings. First, the PMI risk prediction models were derived from high-risk patients, as this accurate risk prediction is of highest medical need. Future studies need to evaluate the performance of these models in low-risk patients. Second, there is no universally accepted absolute hs-cTnT/I increase above preoperative concentrations for the definition of PMI. The definition used in this study (absolute increase of the 99th percentile) has been shown to result in prognostically highly relevant events and can be easily generalized to other hs-cTnT/I assays.^23^ However, future studies need to evaluate the performance of these models with possible alternative PMI definitions.

## Conclusions

While currently available preoperative risk scores show poor predictive accuracy for PMI in high-risk patients undergoing noncardiac surgery, cTn adds incremental value above preoperative patient- and procedure-related variables, as well as routine laboratory, and could aid in the prediction of PMI.

Additional BASEL-PMI Investigators and contributors to the Pub-med-listing in PubMed include:

Katharina Rentsch, Esther Seeberger, Silvia Maiorano, Samantha Weder, Jeanne du Fay de Lavallaz, Ketina Arslani, Andreas Buser, Ivo Strebel, Thomas Wolff, Thomas Nestelberger, Philip Haaf, Murat Bilici, Pedro Lopez Ayala Lopez, Luca Koechlin.

## Supplementary material

Supplementary material is available at *European Heart Journal: Acute Cardiovascular Care* online.

## Supplementary Material

zuad090_Supplementary_Data

## Data Availability

Data are available upon reasonable request following an embargo phase until the main results of the BASEL-PMI study are published.
