# Supplementary material for: Prediction of perioperative myocardial infarction/injury in high-risk patients after noncardiac surgery
Source: Eur Heart J Acute Cardiovasc Care. 2023 Aug 7;12(11):729–39. doi: 10.1093/ehjacc/zuad090 (PMC10655147; doi:10.1093/ehjacc/zuad090)
Supplement: zuad090_Supplementary_Data [file zuad090_supplementary_data.docx]

Supplemental Data

**Prediction of perioperative myocardial infarction/injury in high-risk patients after noncardiac surgery**

Rebecca Meister, MD^a#^; Christian Puelacher, MD, PhD^a,b#^; Noemi Glarner, MD^a^; PhDc^a^; Danielle Menosi Gualandro, MD, PhD^a,c^; Henrik A Andersson, PhD^d^; Mirjam Pargger, MD, PhDc^a^; Gabrielle Huré, MSc^a^; Georgiana Virant, MS^a^; Daniel Bolliger, MD Prof^e^; Andreas Lampart, MD^e^; Luzius Steiner, MD Prof^e,f^; Reka Hidvegi, MD^a,g^, Giovanna Lurati Buse, MD, MSc^h^; Christoph Kindler, MD Prof^i^; Lorenz Gürke, MD Prof^k^; Edin Mujagic, MD^k^; Stefan Schaeren, MD Prof^l^; Martin Clauss; MD^m^; Didier Lardinois, MD Prof^n^; Angelika Hammerer-Lercher, MD^o^; Michelle Chew, MD Prof^d^; Christian Mueller, MD Prof^a^, for the BASEL-PMI Investigators^§^

^a^Cardiovascular Research Institute Basel (CRIB) and Department of Cardiology, University Hospital Basel, University of Basel, Switzerland; ^b^Department of Internal Medicine, University Hospital Basel, University of Basel, Basel; ^c^Interdisciplinary Medicine in Cardiology Unit, Cardiology Department, Heart Institute (InCor), University of Sao Paulo Medical School, Brazil; ^d^Department of Anaesthesiology and Intensive Care Medicine, Linköping University Hospital, Sweden; ^e^Department of Anaesthesiology, University Hospital Basel, University of Basel, Basel; ^f^Department of Clinical Research, University Basel, Basel; ^g^Department of Anaesthesiology, Cantonal Hospital St. Gallen, St. Gallen; ^h^Department of Anaesthesiology, University Hospital Dusseldorf, Dusseldorf; ^i^Department of Anaesthesiology, Cantonal Hospital Aarau, Aarau; ^k^Department of Vascular Surgery, University Hospital Basel, University Basel, Basel; ^l^Department of Spinal Surgery, University Hospital Basel, University Basel, Basel; ^m^Department of Orthopedics and Center of Musculoskeletal Infections, University Hospital Basel, University Basel, Basel; ^n^Department of Thoracic Surgery, University Hospital Basel, University of Basel, Basel; ^o^Department of Laboratory Medicine, Cantonal Hospital Aarau, Aarau

^#^both authors have contributed equally and shall be considered co-first author

# **Examples for perioperative troponin measurements and definition of perioperative myocardial infarction/injury**

PMI was prospectively defined as an absolute increase of ≥14 ng/L for hs-cTnT and ≥45 ng/L for s-cTnI (the 99^th^ percentile of each assays) above the preoperative concentration (or between two postoperative concentrations if the preoperative measurement was missing) within three days following surgery, without need for presence of additional signs and symptoms, as recommended by current guidelines.^1^

# Therefore, when using hs-cTnT PMI was diagnosed with a preoperative level of e.g. 5ng/l to postoperative levels of 21ng/l (delta of 16ng/l), or from preoperative 20ng/l to postoperative 34ng/l (delta 14ng/l). On the contrary, a patient with preoperative hs-cTnT of 20 ng/L rising to 30 ng/L was NOT diagnosed as PMI (delta 10ng/l). Also, absolute increases of ≥+14 ng/L following postoperative day 3 were not considered to be PMI, but collected as separate follow-up events, e.g. as acute myocardial infarction on day 6.

# Measures were taken to not adjudicate PMI in cases of cardiac acute events taking place preoperatively. Therefore PMI was not adjudicated if there were missing preoperative cTn concentration and postoperative cTn concentrations elevated above the 99^th^ percentile without a dynamic change OR preoperatively elevated cTn values which were falling perioperatively, e.g. following preoperative type 1 myocardial infarction or recent cardiac surgery (defined as drop of >-50ng/l hs-cTnT or >-200ng/l s-cTnI). Events might have begun preoperatively (e.g. cardiac decompensation in emergent surgery), but PMI was adjudicated only if there was an exacerbation perioperatively with acute perioperative increase in cTn.

# **Variables**

## Body-mass-index (BMI) was categorized into underweight, normal weight and overweight according to the definition of the World Health Organization (WHO).^2^

Hypertension was defined as known history of hypertonia.

Diabetes was defined as known history of diabetes divided into insulin-dependent and non-insulin-dependent diabetes.

## Coronary artery disease and previous myocardial infarction were defined as history of coronary artery disease, history of acute myocardial infarction, finding of stenosis on coronary angiogram, or positive stress testing.

Congestive heart failure was defined as history of congestive heart failure, left ventricular ejection fraction ≤40%, or diastolic dysfunction grade II or higher with elevated B-type natriuretic peptide irrespective of ejection fraction.

Atrial fibrillation was defined as history of paroxysmal, persistent or permanent atrial fibrillation occurring more than once, or atrial fibrillation on preoperative ECG.

Peripheral artery disease was defined as history of peripheral artery disease, known carotid stenosis, or arterial vascular surgery for aortic aneurysm.

Stroke or transient ischemic attack was defined as history of acute new focal neurological deficit judged by treating physicians to be of vascular cause lasting >24 hours.

Chronic kidney disease was defined as known renal failure classified by Chronic Kidney Disease Epidemiology Collaboration (CKD-EPI) Formula for GFR.^3^

Functional capacity was defined by metabolic equivalents of task and is classified as normal with a cut-off above 4. We used self-reported functional capacity estimated by the ability of climbing two flights of stairs to approximate 4 metabolic equivalents

## The revised cardiac risk index (RCRI) was used as defined by Lee et al.^4^

| Revised cardiac risk index (RCRI) |
| --- |
| 1. High-risk type of surgery (suprainguinal vascular, intraperitoneal or intrathoracic) |
| 2. Ischemic heart disease |
| 3. History of congestive heart failure |
| 4. History of cerebrovascular disease |
| 5. Insulin therapy for diabetes |
| 6. Preoperative serum creatinine >2.0 mg/dL |

**Supplemental Table 1:** Risk for cardiac death, nonfatal myocardial infarction and nonfatal cardiac arrest predicted by the revised cardiac risk index (RCRI): 0 predictors = 0.4%, 1 predictor = 0.9%, 2 predictors = 7%, ≥ 3 predictors = 11%.

The ASA risk score was defined according to the American Society of Anesthesiology (ASA) Physical Status Classification.^5^

| ASA Class | |
| --- | --- |
| I | A normal healthy patient |
| II | A patient with mild systemic disease |
| III | A patient with severe systemic disease |
| IV | A patient with severe systemic disease that is a constant threat to life |
| V | A moribund patient who is not expected to survive without the operation |
| VI | A declared brain-dead patient whose organs are being removed for donor purposes |

**Supplemental Table 2:** Physical status classification according to the American Society of Anesthesiology (ASA) to assess a patient’s perioperative risk.

|  | **All patients  (n = 9694; 100%)** | **Preoperative routine laboratory available  (n = 8329; 85.9%)** | **Preoperative routine laboratory not available  (n = 1365; 14.1%)** | **P value** |
| --- | --- | --- | --- | --- |
| Age, years, median (IQR) | 74 (68-79) | 74 (69-79) | 73 (68-79) | 0.152 |
| Male, sex, n (%) | 5446 (56.2%) | 4702 (56.5%) | 744 (54.5%) | 0.179 |
| BMI, kg/m², n (%) |  |  |  | 0.021 |
| < 18.5 kg/m² | 194 (2.1%) | 180 (2.2%) | 14 (1.1%) |  |
| 18.5 - 24.9 kg/m² | 3713 (39.3%) | 3221 (39.4%) | 492 (38.5%) |  |
| ≥ 25 kg/m² | 5537 (58.6%) | 4764 (58.3%) | 773 (60.4%) |  |
| **Risk factors, n (%)** |  |  |  |  |
| Hypertension | 6405 (66.1%) | 5537 (66.5%) | 868 (63.6%) | 0.037 |
| Diabetes |  |  |  | <0.001 |
| NIDDM | 1442 (14.9%) | 1265 (15.2%) | 177 (13.0%) |  |
| IDDM | 893 (9.2%) | 806 (9.7%) | 87 (6.4%) |  |
| **Medical history, n (%)** |  |  |  |  |
| Coronary artery disease | 2839 (29.3%) | 2491 (29.9%) | 348 (25.5%) | 0.001 |
| Previous myocardial infarction | 1447 (14.9%) | 1270 (15.2%) | 177 (13.0%) | 0.028 |
| Congestive heart failure | 1093 (11.3%) | 996 (12.0%) | 97 (7.1%) | <0.001 |
| Atrial fibrillation | 1778 (18.3%) | 1589 (19.1%) | 189 (13.8%) | <0.001 |
| Peripheral artery disease | 1969 (20.3%) | 1750 (21.0%) | 219 (16.0%) | <0.001 |
| Previous stroke or TIA | 723 (7.5%) | 710 (8.5%) | 13 (1.0%) | <0.001 |
| Chronic kidney disease |  |  |  | <0.001 |
| CKD I-II° | 3182 (32.8%) | 2779 (33.4%) | 403 (29.5%) |  |
| CKD III+° | 1298 (13.4%) | 1271 (15.3%) | 27 (2.0%) |  |
| Dialysis-dependent CKD | 192 (2.0%) | 164 (2.0%) | 28 (2.1%) |  |
| Functional capacity > 4 METS | 5698 (59.4%) | 4581 (55.3%) | 1117 (85.2%) | <0.001 |
| Surgery due to a malign tumor | 1592 (16.4%) | 1470 (17.6%) | 122 (8.9%) | <0.001 |
| **Risk of Surgery, n (%)** |  |  |  | <0.001 |
| Low (<1%) | 2797 (28.9%) | 2497 (30.0%) | 300 (22.0%) |  |
| Intermediate (1-5%) | 5781 (59.6%) | 4844 (58.2%) | 937 (68.6%) |  |
| High (>5%) | 1116 (11.5%) | 988 (11.9%) | 128 (9.4%) |  |
| **Time of Surgery, n (%)** |  |  |  | <0.001 |
| Elective | 7105 (73.7%) | 6151 (73.9%) | 954 (69.9%) |  |
| Emergency < 24h | 1056 (10.9%) | 763 (9.2%) | 293 (21.5%) |  |
| Emergency > 24h | 1533 (15.8%) | 1415 (17.0%) | 118 (8.6%) |  |
| **Other parameters, median (IQR)** |  |  |  |  |
| Planned duration of surgery, min | 135 (105-165) | 135 (105-165) | 105 (105-135) | <0.001 |
| **Risk Score ASA, n (%)** |  |  |  | <0.001 |
| 1 | 105 (1.1%) | 81 (1.0%) | 24 (1.8%) |  |
| 2 | 3133 (32.7%) | 2468 (29.8%) | 665 (50.7%) |  |
| 3 | 5735 (59.8%) | 5148 (62.2%) | 587 (44.8%) |  |
| 4 | 609 (6.4%) | 574 (6.9%) | 35 (2.7%) |  |
| 5 | 6 (0.1%) | 6 (0.1%) | 0 (0.0%) |  |
| **RCRI, n (%)** |  |  |  | <0.001 |
| I | 4265 (44.0%) | 3590 (43.1%) | 675 (49.5%) |  |
| II | 3253 (33.6%) | 2762 (33.2%) | 491 (36.0%) |  |
| III | 1464 (15.1%) | 1309 (15.7%) | 155 (11.4%) |  |
| IV | 712 (7.3%) | 668 (8.0%) | 44 (3.2%) |  |
| **PMI, n (%)** | 1345 (13.9%) | 1231 (14.8%) | 114 (8.4%) | <0.001 |

# **Supplemental Table 3. Baseline Characteristics of patients with preoperative routinely available laboratory and unavailable preoperative routinely laboratory.** BMI = body-mass-index; NIDDM = non insulin dependent diabetes mellitus; IDDM = insulin dependent diabetes mellitus; TIA = transient ischemic attack; CKD = chronic kidney disease; MET = metabolic equivalent of task; ASA = American Society of Anesthesiology; RCRI = Revised Cardiac Risk Index; PMI = perioperative myocardial infarction/injury

| **Variables** | **OR** | **95% CI for OR** | |
| --- | --- | --- | --- |
|  |  | Lower CI | Upper CI |
| **Age, years** | 1.020 | 1.010 | 1.030 |
| **BMI 18.5-24.9 kg/m^2^** |  |  |  |
| **BMI < 18.5 kg/m^2^** | 1.266 | 0.890 | 1.801 |
| **BMI ≥ 25 kg/m^2^** | 0.755 | 0.652 | 0.875 |
| **No Diabetes** |  |  |  |
| **NIDDM** | 1.034 | 0.850 | 1.258 |
| **IDDM** | 1.571 | 1.264 | 1.952 |
| **No history of CAD or MI** |  |  |  |
| **CAD without MI** | 1.490 | 1.230 | 1.804 |
| **CAD with MI** | 2.073 | 1.730 | 2.485 |
| **History of heart failure** | 1.318 | 1.083 | 1.605 |
| **AF** | 1.313 | 1.107 | 1.557 |
| **No CKD** |  |  |  |
| **CKD I-II°** | 1.348 | 1.138 | 1.598 |
| **CKD III°+** | 1.799 | 1.473 | 2.197 |
| **Dialysis-dependent CKD** | 4.285 | 2.893 | 6.346 |
| **MET >4** | 0.550 | 0.474 | 0.639 |
| **Low risk surgery ESC <1%** |  |  |  |
| **Intermediate risk surgery ESC 1%-5%** | 1.582 | 1.325 | 1.889 |
| **High risk surgery ESC >5%** | 1.834 | 1.447 | 2.324 |
| **Elective surgery** |  |  |  |
| **Urgent surgery <24h** | 2.018 | 1.604 | 2.540 |
| **Urgent surgery >24h** | 1.418 | 1.180 | 1.705 |
| **Planned duration of surgery, min** | 1.005 | 1.004 | 1.007 |

**Supplemental Table 4. Logistic regression predicting perioperative myocardial injury using clinical information (Model 1).**OR = odds ratio; CI = confidence interval; BMI = body-mass-index; NIDDM = non insulin dependent diabetes mellitus; IDDM = insulin dependent diabetes mellitus; CAD = coronary artery disease; MI = myocardial infarction; AF = atrial fibrillation; CKD = chronic kidney disease; MET = metabolic equivalent of task; ESC = European Society of Cardiology

| **Variables** | **OR** | **95% CI for OR** | |
| --- | --- | --- | --- |
|  |  | Lower CI | Upper CI |
| **Age, years** | 1.022 | 1.012 | 1.033 |
| **BMI 18.5-24.9 kg/m^2^** |  |  |  |
| **BMI < 18.5 kg/m^2^** | 1.190 | 0.834 | 1.698 |
| **BMI ≥ 25 kg/m^2^** | 0.819 | 0.705 | 0.951 |
| **No Diabetes** |  |  |  |
| **NIDDM** | 0.962 | 0.789 | 1.173 |
| **IDDM** | 1.385 | 1.111 | 1.726 |
| **No history of CAD or MI** |  |  |  |
| **CAD without MI** | 1.455 | 1.199 | 1.766 |
| **CAD with MI** | 2.033 | 1.692 | 2.442 |
| **History of heart failure** | 1.242 | 1.019 | 1.515 |
| **AF** | 1.223 | 1.028 | 1.454 |
| **No CKD** |  |  |  |
| **CKD I-II°** | 1.300 | 1.095 | 1.545 |
| **CKD III°+** | 1.621 | 1.323 | 1.987 |
| **Dialysis-dependent CKD** | 3.834 | 2.581 | 5.695 |
| **MET >4** | 0.650 | 0.557 | 0.758 |
| **Low risk surgery ESC <1%** |  |  |  |
| **Intermediate risk surgery ESC 1%-5%** | 1.687 | 1.409 | 2.020 |
| **High risk surgery ESC >5%** | 1.806 | 1.421 | 2.296 |
| **Elective surgery** |  |  |  |
| **Urgent surgery <24h** | 1.781 | 1.400 | 2.266 |
| **Urgent surgery >24h** | 1.169 | 0.967 | 1.414 |
| **Planned duration of surgery, min** | 1.006 | 1.005 | 1.007 |
| **Hb normal, g/l** |  |  |  |
| **Mild to moderate anemia** | 2.089 | 1.780 | 2.452 |
| **Severe anemia** | 2.322 | 1.624 | 3.319 |
| **Tc normal, x10S9/L** |  |  |  |
| **Thrombopenia** | 1.208 | 0.951 | 1.535 |
| **Thrombocytosis** | 1.613 | 1.254 | 2.075 |
| **Lc normal, x10S9/L** |  |  |  |
| **Leukopenia** | 0.733 | 0.455 | 1.179 |
| **Leukocytosis** | 1.328 | 1.118 | 1.578 |
| **Sodium normal, mmol/L** |  |  |  |
| **Hyponatremia** | 1.098 | 0.876 | 1.376 |
| **Hypernatremia** | 1.685 | 1.126 | 2.522 |

**Supplemental Table 5.** **Logistic regression predicting perioperative myocardial injury using clinical information and routinely available preoperative laboratory values (Model 2).**

OR = odds ratio; CI = confidence interval; BMI = body-mass-index; NIDDM = non insulin dependent diabetes mellitus; IDDM = insulin dependent diabetes mellitus; CAD = coronary artery disease; MI = myocardial infarction; AF = atrial fibrillation; CKD = chronic kidney disease; MET = metabolic equivalent of task; ESC = European Society of Cardiology; Hb = hemoglobin; Tc = thrombocyte; Lc = leukocyte

| **Variables** | **OR** | **95% CI for OR** | |
| --- | --- | --- | --- |
|  |  | Lower CI | Upper CI |
| **Age, years** | 1.011 | 1.001 | 1.021 |
| **BMI 18.5-24.9 kg/m^2^** |  |  |  |
| **BMI < 18.5 kg/m^2^** | 1.192 | 0.829 | 1.716 |
| **BMI ≥ 25 kg/m^2^** | 0.804 | 0.692 | 0.934 |
| **No history of CAD or MI** |  |  |  |
| **CAD without MI** | 1.353 | 1.116 | 1.641 |
| **CAD with MI** | 1.776 | 1.476 | 2.137 |
| **MET >4** | 0.780 | 0.666 | 0.912 |
| **Low risk surgery ESC <1%** |  |  |  |
| **Intermediate risk surgery ESC 1%-5%** | 1.770 | 1.470 | 2.131 |
| **High risk surgery ESC >5%** | 1.804 | 1.412 | 2.304 |
| **Elective surgery** |  |  |  |
| **Urgent surgery <24h** | 1.724 | 1.362 | 2.182 |
| **Urgent surgery >24h** | 1.106 | 0.918 | 1.333 |
| **Planned duration of surgery, min** | 1.007 | 1.005 | 1.008 |
| **Hb normal, g/l** |  |  |  |
| **Mild to moderate anemia** | 1.538 | 1.302 | 1.818 |
| **Severe anemia** | 1.261 | 0.866 | 1.836 |
| **Tc normal, x10S9/L** |  |  |  |
| **Thrombopenia** | 1.061 | 0.833 | 1.351 |
| **Thrombocytosis** | 1.583 | 1.230 | 2.037 |
| **Hs-cTnT (ln), ng/L** | 2.382 | 2.164 | 2.622 |

**Supplemental Table 6.** **Logistic regression predicting perioperative myocardial injury using clinical information, routinely available preoperative laboratory values and preoperative hs-cTnT (Model 3).**

Hs-cTnT = high-sensitivity cardiac troponin T; OR = odds ratio; CI = confidence interval; BMI = body-mass-index; NIDDM = non insulin dependent diabetes mellitus; IDDM = insulin dependent diabetes mellitus; CAD = coronary artery disease; MI = myocardial infarction; MET = metabolic equivalent of task; ESC = European Society of Cardiology; Hb = hemoglobin; Tc = thrombocyte

|  | **R** | **R Square** | **Adjusted R Square** | **Std. Error of the Estimate** | **Akaike Information Criterion** |
| --- | --- | --- | --- | --- | --- |
| **Model 1** | 0.288 | 0.083 | 0.082 | 0.344 | -14792.249 |
| **Model 2** | 0.319 | 0.102 | 0.100 | 0.341 | -14927.429 |
| **Model 3** | 0.365 | 0.133 | 0.132 | 0.335 | -15185.077 |
| **ASA Classification** | 0.177 | 0.031 | 0.031 | 0.354 | -14430.038 |
| **RCRI** | 0.198 | 0.039 | 0.039 | 0.352 | -14487.415 |

**Supplemental Table 7. Akaike Information Criterion:**AIC to compare the different multivariable regression models for prediction of PMI.
ASA Classification = American Society of Anesthesiology Physical Status Classification; RCRI = Revised Cardiac Risk Index; AIC = Akaike Information Criterion; PMI = perioperative myocardial infarction/injury

|  | **All patients (n = 7910; 100%)** | **Derivation (n=6944; 87.8%)** | **Recalibration (n=966; 12.2%)** | **P value** |
| --- | --- | --- | --- | --- |
| Age, years, median (IQR) | 74 (69-79) | 74 (69-79) | 74 (69-81) | <0.001 |
| Male, sex, n (%) | 4472 (56.5%) | 3937 (56.7%) | 535 (55.4%) | 0.440 |
| BMI, kg/m², n (%) |  |  |  | 0.003 |
| < 18.5 kg/m² | 266 (3.4%) | 242 (3.5%) | 24 (2.5%) |  |
| 18.5 - 24.9 kg/m² | 3038 (38.4%) | 2706 (39.0%) | 332 (34.4%) |  |
| ≥ 25 kg/m² | 4606 (58.2%) | 3996 (57.5%) | 610 (63.1%) |  |
| **Risk factors, n (%)** |  |  |  |  |
| Hypertension | 5312 (67.2%) | 4603 (66.3%) | 709 (73.4%) | <0.001 |
| Diabetes |  |  |  | 0.025 |
| NIDDM | 1199 (15.2%) | 1064 (15.3%) | 135 (14.0%) |  |
| IDDM | 780 (9.9%) | 662 (9.5%) | 118 (12.2%) |  |
| **Medical history, n (%)** |  |  |  |  |
| Coronary artery disease | 2394 (30.3%) | 2118 (30.5%) | 276 (28.6%) | 0.221 |
| Previous myocardial infarction | 1214 (15.3%) | 1075 (15.5%) | 139 (14.4%) | 0.378 |
| Congestive heart failure |  |  |  |  |
| Preserved LVEF (> 50%) | 294 (3.7%) | 253 (3.6%) | 41 (4.2%) | 0.006 |
| Medium LVEF (40-50%) | 288 (3.6%) | 247 (3.6%) | 41 (4.2%) | 0.006 |
| Reduced LVEF (<40%) | 323 (4.1%) | 302 (4.3%) | 21 (2.2%) | 0.006 |
| LVEF not graduated | 45 (0.6%) | 36 (0.5%) | 9 (0.9%) | 0.006 |
| Atrial fibrillation | 1497 (18.9%) | 1287 (18.5%) | 210 (21.7%) | 0.017 |
| Peripheral artery disease | 1685 (21.3%) | 1406 (20.2%) | 279 (28.9%) | <0.001 |
| Previous stroke or TIA | 684 (8.6%) | 684 (9.9%) | 0 (0.0%) | <0.001 |
| Chronic kidney disease |  |  |  | <0.001 |
| CKD I-II° | 2656 (33.6%) | 2246 (32.3%) | 410 (42.2%) |  |
| CKD III+° | 1211 (15.3%) | 1023 (14.7%) | 188 (19.5%) |  |
| Dialysis-dependent CKD | 154 (1.9%) | 132 (1.9%) | 22 (2.3%) |  |
| Functional capacity > 4 METS | 4388 (55.5%) | 3609 (52.0%) | 779 (80.6%) | <0.001 |
| **Type of Surgery, n (%)** |  |  |  | <0.001 |
| Orthopedic | 1823 (23.0%) | 1419 (20.4%) | 404 (41.8%) |  |
| Spine | 1173 (14.8%) | 1173 (16.9%) | 0 (0.0%) |  |
| Thoracic | 644 (8.1%) | 644 (9.3%) | 0 (0.0%) |  |
| Trauma | 611 (7.7%) | 526 (7.6%) | 85 (8.8%) |  |
| Urologic | 1131 (14.3%) | 1131 (16.3%) | 0 (0.0%) |  |
| Visceral | 1094 (13.8%) | 856 (12.3%) | 238 (24.6%) |  |
| Vascular | 1272 (16.1%) | 1036 (14.9%) | 236 (24.4%) |  |
| Other | 162 (2.0%) | 159 (2.3%) | 3 (0.3%) |  |
| **Risk of Surgery, n (%)** |  |  |  | <0.001 |
| Low (<1%) | 2357 (29.8%) | 2255 (32.5%) | 102 (10.6%) |  |
| Intermediate (1-5%) | 4616 (58.4%) | 3908 (56.3%) | 708 (73.3%) |  |
| High (>5%) | 937 (11.8%) | 781 (11.2%) | 156 (16.1%) |  |
| **Time of Surgery, n (%)** |  |  |  | <0.001 |
| Elective | 5871 (74.2%) | 5055 (72.8%) | 816 (84.5%) |  |
| Emergency < 24h | 694 (8.8%) | 648 (9.3%) | 46 (4.8%) |  |
| Emergency > 24h | 1345 (17.0%) | 1241 (17.9%) | 104 (10.8%) |  |
| **Other parameters, median (IQR)** |  |  |  |  |
| Preoperative SBP, mmHg | 136 (123-150) | 136 (123-150) | 136 (121-152) | 0.822 |
| Planned duration of surgery, min | 135 (105-165) | 135 (105-165) | NA | NA |
| **Risk Score ASA, n (%)** |  |  |  | <0.001 |
| 1 | 81 (1.0%) | 72 (1.0%) | 9 (0.9%) |  |
| 2 | 2343 (29.6%) | 1988 (28.6%) | 355 (36.7%) |  |
| 3 | 4936 (62.4%) | 4371 (62.9%) | 565 (58.5%) |  |
| 4 | 546 (6.9%) | 509 (7.3%) | 37 (3.8%) |  |
| 5 | 4 (0.1%) | 4 (0.1%) | 0 (0.0%) |  |
| **RCRI, n (%)** |  |  |  | 0.161 |
| I | 3379 (42.7%) | 2969 (42.8%) | 410 (42.4%) |  |
| II | 2630 (33.2%) | 2285 (32.9%) | 345 (35.7%) |  |
| III | 1257 (15.9%) | 1124 (16.2%) | 133 (13.8%) |  |
| IV | 644 (8.1%) | 566 (8.2%) | 78 (8.1%) |  |
| **Biochemistry, median (IQR)** |  |  |  |  |
| Hemoglobin, g/L | 126 (108-140) | 126 (109-140) | 123 (105-138) | 0.001 |
| Platelets, x10S9/L | 242 (194-305) | 242 (193-306) | 243 (196-301) | 0.781 |
| Leukocytes, x10S9/L | 7.5 (6.1-9.5) | 7.5 (6.1-9.4) | 7.7 (6.2-9.6) | 0.032 |
| Creatinine, µmol/L | 82.0 (67.0-105) | 81.0 (67.0-105) | 84.0 (68.0-110) | 0.004 |
| Sodium, mmol/L | 140 (138-141) | 140 (137-141) | 140 (138-141) | 0.191 |
| Potassium, mmol/L | 4.1 (3.8-4.4) | 4.1 (3.8-4.4) | 3.9 (3.7-4.2) | <0.001 |
| Preoperative hs-cTnT, ng/L | 14.0 (9.0-26.0) | 14.0 (9.0-26.0) | NA | NA |
| Preoperative s-cTnI, ng/L | 14.9 (14.9-14.9) | NA | 14.9 (14.9-14.9) | NA |
| **PMI, n (%)** | 1171 (14.8%) | 1058 (15.2%) | 113 (11.7%) | 0.004 |

# **Supplemental Table 8. Baseline characteristics of the derivation and recalibration cohort** BMI = body-mass-index; NIDDM = non insulin dependent diabetes mellitus; IDDM = insulin dependent diabetes mellitus; LVEF = left ventricular ejection fraction; TIA = transient ischemic attack; CKD = chronic kidney disease; MET = metabolic equivalent of task; SBP = systolic blood pressure; NA = not available; ASA = American Society of Anesthesiology; RCRI = Revised Cardiac Risk Index; hs-cTnT = high-sensitivity cardiac troponin T; s-cTnI = sensitive cardiac troponin I; PMI = perioperative myocardial infarction/injury

#

| **Variables, OR (95% CI for OR)** | **Model 1** | **Model 2** | **Model 3** |
| --- | --- | --- | --- |
| Age, years | 1.050 (1.020-1.081) | 1.052 (1.021-1.083) | 1.057 (1.029-1.086) |
| BMI 18.5-24.9 kg/m^2^ |  |  |  |
| BMI < 18.5 kg/m^2^ | 0.972 (0.254-3.721) | 0.918 (0.227-3.707) | 1.044 (0.265-4.113) |
| BMI ≥ 25 kg/m^2^ | 1.148 (0.729-1.807) | 1.137 (0.710-1.819) | 1.332 (0.837-2.117) |
| No Diabetes |  |  |  |
| NIDDM | 0.760 (0.400-1.443) | 0.749 (0.390-1.438) |  |
| IDDM | 0.861 (0.440-1.685) | 0.886 (0.448-1.749) |  |
| No history of CAD or MI |  |  |  |
| CAD without MI | 1.475 (0.827-2.632) | 1.449 (0.804-2.610) | 1.234 (0.676-2.250) |
| CAD with MI | 2.570 (1.488-4.440) | 2.515 (1.447-4.373) | 2.536 (1.483-4.337) |
| History of heart failure | 1.448 (0.824-2.545) | 1.486 (0.837-2.637) |  |
| AF | 1.219 (0.750-1.982) | 1.176 (0.714-1.938) |  |
| No CKD |  |  |  |
| CKD I-II° | 1.387 (0.809-2.379) | 1.386 (0.806-2.383) |  |
| CKD III°+ | 1.538 (0.802-2.946) | 1.609 (0.834-3.105) |  |
| Dialysis-dependent CKD | 1.196 (0.297-4.813) | 1.245 (0.302-5.129) |  |
| MET >4 | 0.687 (0.418-1.129) | 0.681 (0.407-1.140) | 0.745 (0.446-1.246) |
| Low risk surgery ESC <1% |  |  |  |
| Intermediate risk surgery ESC 1%-5% | 2.870 (1.112-7.403) | 3.255 (1.217-8.708) | 3.532 (1.293-9.646) |
| High risk surgery ESC >5% | 4.598 (1.647-12.835) | 4.792 (1.675-13.711) | 5.480 (1.886-15.925) |
| Elective surgery |  |  |  |
| Urgent surgery <24h | 0.684 (0.227-2.061) | 0.651 (0.208-2.037) | 0.634 (0.212-1.897) |
| Urgent surgery >24h | 0.846 (0.436-1.644) | 0.845 (0.424-1.682) | 0.647 (0.320-1.307) |
| Hb normal, g/L |  |  |  |
| Mild to moderate anemia |  | 0.876 (0.543-1.414) | 0.952 (0.598-1.517) |
| Severe anemia |  | 1.959 (0.658-5.829) | 1.519 (0.500-4.621) |
| Tc normal, x10S9/L |  |  |  |
| Thrombopenia |  | 1.319 (0.646-2.695) | 1.258 (0.612-2.589) |
| Thrombocytosis |  | 1.567 (0.583-4.212) | 1.444 (0.552-3.778) |
| Lc normal, x10S9/L |  |  |  |
| Leukopenia |  | 0.875 (0.176-4.346) |  |
| Leukocytosis |  | 1.136 (0.674-1.914) |  |
| Sodium normal, mmol/L |  |  |  |
| Hyponatremia |  | 0.715 (0.271-1.891) |  |
| Hypernatremia |  | 0.303 (0.063-1.454) |  |
| S-cTnI (ln), ng/L |  |  | 2.103 (1.554-2.847) |

**Supplemental Table 9. Logistic regression models for prediction of perioperative myocardial injury tested in the recalibration cohort.**Model 1 used clinical information alone, Model 2 clinical information with additional preoperative routinely available laboratory values and Model 3 clinical information with additional preoperative routinely available laboratory values and preoperative s-cTnI for prediction of PMI.

S-cTnI = sensitive cardiac troponin I; PMI = perioperative myocardial infarction/injury; OR = odds ratio; CI = confidence interval; BMI = body-mass-index; NIDDM = non insulin dependent diabetes mellitus; IDDM = insulin dependent diabetes mellitus; CAD = coronary artery disease; MI = myocardial infarction; AF = atrial fibrillation; CKD = chronic kidney disease; MET = metabolic equivalent of task; ESC = European Society of Cardiology; Hb = hemoglobin; Tc = thrombocyte; Lc = leukocyte

|  | **All patients  (n = 722; 100%)** | **PMI  (n = 91; 12.6%)** | **No PMI (n = 631; 87.4%)** | **P value** |
| --- | --- | --- | --- | --- |
| Age, years, median (IQR) | 70 (64-75) | 72 (67-76) | 70 (63-75) | 0.076 |
| Male, sex, n (%) | 411 (56.9%) | 62 (68.1%) | 349 (55.3%) | 0.023 |
| BMI, kg/m², n (%) |  |  |  | 0.806 |
| < 18.5 kg/m² | 11 (1.5%) | 1 (1.1%) | 10 (1.6%) |  |
| 18.5 - 24.9 kg/m² | 290 (40.2%) | 36 (39.6%) | 254 (40.3%) |  |
| ≥ 25 kg/m² | 421 (58.3%) | 54 (59.3%) | 367 (58.2%) |  |
| **Risk factors, n (%)** |  |  |  |  |
| Hypertension | 376 (52.1%) | 36 (39.6%) | 340 (53.9%) | 0.013 |
| IDDM | 63 (8.7%) | 13 (14.3%) | 50 (7.9%) | 0.070 |
| **Medical history, n (%)** |  |  |  |  |
| Coronary artery disease | 91 (12.6%) | 22 (24.2%) | 69 (10.9%) | <0.001 |
| Congestive heart failure | 25 (3.5%) | 10 (11.0%) | 15 (2.4%) | <0.001 |
| Atrial fibrillation | 69 (9.6%) | 10 (11.0%) | 59 (9.4%) | 0.571 |
| Previous stroke or TIA | 52 (7.2%) | 4 (4.4%) | 48 (7.6%) | 0.384 |
| Chronic kidney disease | 8 (1.1%) | 5 (5.5%) | 3 (0.5%) | <0.001 |
| Functional capacity > 4 METS | 419 (58.0%) | 45 (49.5%) | 374 (59.3%) | 0.088 |
| **Type of Surgery, n (%)** |  |  |  | 0.337 |
| Upper gastrointestinal | 92 (12.7%) | 11 (12.1%) | 81 (12.8%) |  |
| Hepatobiliary | 212 (29.4%) | 21 (23.1%) | 191 (30.3%) |  |
| Pancreas | 176 (24.4%) | 30 (33.0%) | 146 (23.1%) |  |
| Colorectal | 118 (16.3%) | 14 (15.4%) | 104 (16.5%) |  |
| Urology (not renal) | 34 (4.7%) | 2 (2.2%) | 32 (5.1%) |  |
| Renal | 71 (9.8%) | 12 (13.2%) | 59 (9.4%) |  |
| Gynaecology | 6 (0.8%) | 0 (0%) | 6 (1.0%) |  |
| Other | 13 (1.8%) | 1 (1.1%) | 12 (1.9%) |  |
| **Risk of Surgery (ESC guidelines), n (%)** | |  |  | 0.470 |
| Low | 2 (0.3%) | 0 (0%) | 2 (0.3%) |  |
| Intermediate | 260 (36.0%) | 30 (33.0%) | 230 (36.5%) |  |
| High | 460 (63.7%) | 61 (67.0%) | 399 (63.2%) |  |
| **Time of Surgery, n (%)** |  |  |  |  |
| Elective | 722 (100%) |  |  |  |
| **Other parameters, median (IQR)** |  |  |  |  |
| Preinduction SBP, mmHg | 146 (132-161) | 149 (134-167) | 145 (132-160) | 0.161 |
| Lenght of surgery, min | 170 (120-280) | 200 (145-315) | 167 (115-271) | 0.005 |
| **Risk Score ASA, n (%)** |  |  |  | 0.008 |
| 1 | 103 (14.3%) | 7 (7.7%) | 96 (15.2%) |  |
| 2 | 384 (53.2%) | 45 (49.5%) | 339 (53.7%) |  |
| 3 | 232 (32.1%) | 38 (41.8%) | 194 (30.7%) |  |
| 4 | 3 (0.4%) | 1 (1.1%) | 2 (0.3%) |  |
| 5 | 0 (0%) | 0 (0%) | 0 (0%) |  |
| **RCRI, n (%)** |  |  |  | <0.001 |
| I | 531 (73.5%) | 51 (56.0%) | 480 (76.1%) |  |
| II | 150 (20.8%) | 27 (29.7%) | 123 (19.5%) |  |
| III | 34 (4.7%) | 12 (13.2%) | 22 (3.5%) |  |
| IV | 7 (1.0%) | 1 (1.1%) | 6 (1.0%) |  |
| **Biochemistry, median (IQR)** |  |  |  |  |
| Preoperative Hemoglobin, g/L | 130 (118-141) | 126 (116-138) | 130 (119-141) | 0.033 |
| Thrombocytes, x10e9/L | 245 (197-294) | 241 (197-287) | 245 (197-295) | 0.665 |
| Preoperative Creatinine, µmol/L | 78 (66-93) | 82 (71-104) | 77 (66-92) | 0.029 |
| Preoperative hs-cTnT, ng/L | 8.8 (5.9-13.5) | 12.2 (7.1-19.4) | 8.5 (5.7-12.7) | <0.001 |

**Supplemental Table 10. Baseline characteristics of the MINSS study cohort.**

MINS = myocardial injury after noncardiac surgery; PMI = perioperative myocardial infarction/injury; IQR = interquartile range; BMI = body-mass-index; IDDM = insulin dependent diabetes mellitus; TIA = transient ischemic attack; MET = metabolic equivalent of task; SBP = systolic blood pressure; ASA = American Society of Anesthesiology; RCRI = Revised Cardiac Risk Index; hs-cTnT = high-sensitivity cardiac troponin T


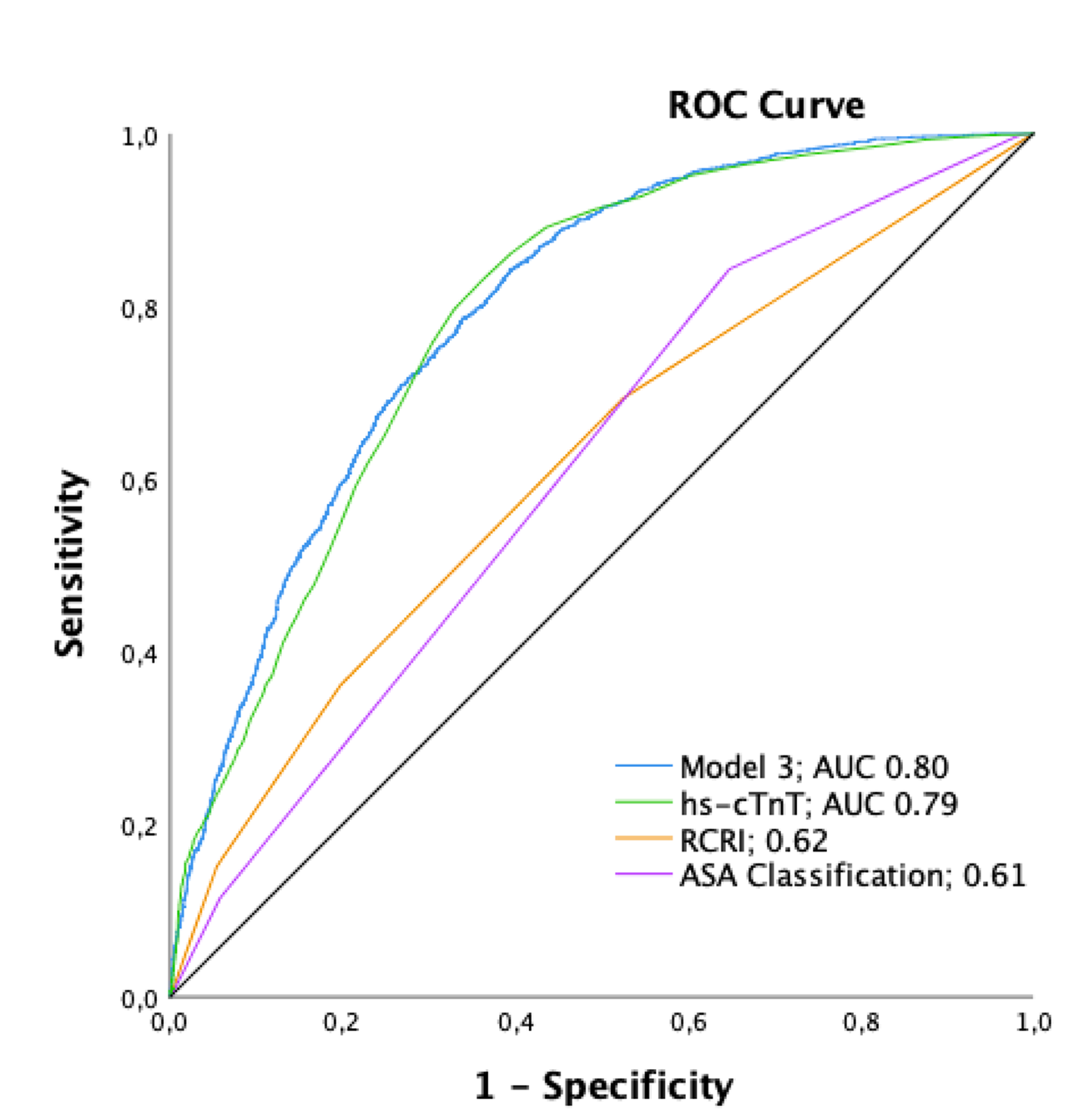


**Figure 1. Accuracy for the prediction of myocardial injury after noncardiac surgery (MINS).**

AUC to compare the diagnostic accuracy of Model 3 with the RCRI, the ASA Classification and hs-cTnT alone

AUC = area under the receiver operating characteristic curve; RCRI = Revised Cardiac Risk Index; ASA = American Society of Anesthesiology

**Supplemental References**

1. Halvorsen S, Mehilli J, Cassese S, et al. 2022 ESC Guidelines on cardiovascular assessment and management of patients undergoing non-cardiac surgery. *Eur Heart J*. Epub ahead of print August 2022. DOI: 10.1093/eurheartj/ehac270.

2. *World Health Organization (2000) Obesity: preventing and managing the global epidemic. Report of a WHO consultation. World Health Organization, Geneva*. Switzerland, 2000.

3. K/DOQI clinical practice guidelines for chronic kidney disease: evaluation, classification, and stratification. *Am J Kidney Dis* 2002; 39: S1-266.

4. Lee TH, Marcantonio ER, Mangione CM, et al. Derivation and prospective validation of a simple index for prediction of cardiac risk of major noncardiac surgery. *Circulation* 1999; 100: 1043–1049.

5. Hurwitz EE, Simon M, Vinta SR, et al. Adding Examples to the ASA-Physical Status Classification Improves Correct Assignment to Patients. *Anesthesiology* 2017; 126: 614–622.
